# SpatialPrompt: spatially aware scalable and accurate tool for spot deconvolution and domain identification in spatial transcriptomics

**DOI:** 10.1038/s42003-024-06349-5

**Published:** 2024-05-25

**Authors:** Asish Kumar Swain, Vrushali Pandit, Jyoti Sharma, Pankaj Yadav

**Affiliations:** 1 Department of Bioscience & Bioengineering, Indian Institute of Technology, Jodhpur, Rajasthan 342030 India; 2 School of Artificial Intelligence and Data Science, Indian Institute of Technology, Jodhpur, Rajasthan 342030 India

**Keywords:** Data integration, Genetic databases

## Abstract

Efficiently mapping of cell types in situ remains a major challenge in spatial transcriptomics. Most spot deconvolution tools ignore spatial coordinate information and perform extremely slow on large datasets. Here, we introduce SpatialPrompt, a spatially aware and scalable tool for spot deconvolution and domain identification. SpatialPrompt integrates gene expression, spatial location, and single-cell RNA sequencing (scRNA-seq) dataset as reference to accurately infer cell-type proportions of spatial spots. SpatialPrompt uses non-negative ridge regression and graph neural network to efficiently capture local microenvironment information. Our extensive benchmarking analysis on Visium, Slide-seq, and MERFISH datasets demonstrated superior performance of SpatialPrompt over 15 existing tools. On mouse hippocampus dataset, SpatialPrompt achieves spot deconvolution and domain identification within 2 minutes for 50,000 spots. Overall, domain identification using SpatialPrompt was 44 to 150 times faster than existing methods. We build a database housing 40 plus curated scRNA-seq datasets for seamless integration with SpatialPrompt for spot deconvolution.

## Abstract (web summary)

The authors present SpatialPrompt, a tool that offers high accuracy and scalability for spot deconvolution and domain identification in spatial transcriptomics, showing superior performance on diverse spatial datasets.

## Introduction

Recent advances in sequencing technologies have enable researchers to sequence and analyse samples up to the single-cell resolution. Single-cell RNA sequencing (scRNA-seq) technologies could reveal the heterogeneity of cell types and cell states, albeit they fail to provide any spatial information^1^. Consequently, several spatially resolved transcriptomics approaches have recently emerged that preserve the spatial information of cells while measuring the gene expression profiles^2–5^. Spatial transcriptomics methods can be broadly classified into two categories, namely, fluorescence in situ hybridisation (FISH)-based and sequencing-based^6^. FISH-based methods allow high-resolution spatial localisation of transcripts but have limited ability to detect large numbers of genes^7,8^. Conversely, sequencing-based methods such as 10X Visium^3^ and ST^5^ can detect thousands of genes but at the cost of low resolution ranging 10–100 µm. In low-resolution spatial techniques, each measured location, referred as spot, usually contains a mixture of cell-types^8^. Therefore, identifying the cell-type distribution at each spot is a major challenge in understanding the complex tissue architectures.

Recently, a series of spot deconvolution tools have been developed for assessing cell-type distribution in the spatial datasets. Majority of these tools leverage the cell-type specific gene signatures from reference scRNA-seq datasets to perform spot deconvolution in the spatial datasets. For instance, SPOTlight^9^ and CARD^10^ use a non-negative matrix factorisation approach to deconvolute the spots based on cell-type specific marker genes from the scRNA-seq reference dataset. In contrast, the RCTD^11^ tool uses a supervised approach that employs a probabilistic model to derive maximum likelihood estimate of the proportions of different cell types. Furthermore, Cell2location^12^, and Stereoscope^13^ tools rely on the assumption that both spatial and scRNA-seq datasets follow a negative binomial distribution. Recently, GraphST^14^ tool used a self-supervised contrasting learning approach, that performs cell type deconvolution by integrating scRNA-seq and spatial dataset using graph autoencoder. SONAR^15^ uses poisson-gamma regression model and geographically weighted regression framework to estimate the spatially informed cell type proportions in the spatial data. Noteworthy, none of these tools, except CARD, GraphST, and SONAR, have exploited the rich spatial information available in the spatial datasets. CARD uses spatial correlation structure of the spatial data using a conditional autoregressive modelling assumption. GraphST uses a graph autoencoder and SONAR uses a poisson-gamma regression model to capture the spatial information in the spatial data. Most tools ignore the impact of the microenvironment and important biological factors useful in the context of tissue arrangement. In a typical spatial tissue arrangement, nearby spots tend to have similar cell-type compositions compared to the distant spots^16,17^. To achieve realistic cell-type inference, SpatialPrompt aims to perform spatially informed spot deconvolution of the spatial datasets while considering the effects of microenvironment and spatial arrangement.

Further, the identification of spatial domains that accurately reflect biological reality remains a challenging task in spatial transcriptomics. Most domain identification methods can be grouped into two categories, namely, non-spatial and spatial-based methods^18^. The non-spatial-based methods such as Seurat^19^ and Scanpy^20^ do not utilise the spatial coordinate information available in the spatial datasets. Most of them are merely an extension of the tools that are commonly used for scRNA-seq datasets. Though spatial-based methods^21,22^ such as STAGATE^23^, SEDR^24^, BayesSpace^21^, GraphST^14^, SPAGCN^22^, and BASS^25^ are more biologically interpretable in identifying spatial domains compared to their non-spatial counterpart, but many of them face scalability issue for large spatial datasets due to rely on computationally intensive algorithms. Many of these methods assume that cells within a given spatial domain are positioned close together^26^. This assumption, however, ignores the fact that distant cells can be part of the same microenvironment^27,28^ and nearby cells like rare cell groups can also be part of two different microenvironments.

Here, we introduce a spatially aware scalable and accurate tool for spot deconvolution and clustering in spatial transcriptomics, hereafter referred to as SpatialPrompt. Our tool utilises a state-of-the-art spatial simulator to generate spatial spots that closely resemble the real spatial data. In addition, by incorporating the spatial information from the real spatial data, SpatialPrompt uses an iterative approach inspired from the message passing layer in the graph neural net^29^ to compute weighted mean expression of the local microenvironment for each spatial spot. The major advantage of utilising spatial information is that SpatialPrompt captures the local microenvironment relation for each spatial spot by assigning higher weightage to the nearer spots. By encoding the spot’s own expression and local weighted mean expression, a concatenated matrix is created that can be used for domain identification in spatial data. An integrated model was constructed for spot deconvolution that combines non-negative ridge regression (NRR) with K-nearest neighbour (KNN) regressor model. This model deconvolute the real spatial spots by incorporating spatial embeddings into the simulated spatial spots. Our tool is highly scalable to perform spot deconvolution and clustering on a large dataset containing thousands of spatial spots within seconds. We demonstrate the sensitivity and accuracy of SpatialPrompt for spot deconvolution and domain prediction using mouse cortex and hippocampus datasets generated from 10X Visium^3^, Slide-seq^4^, MERFISH^30^, and STARmap^31^ platforms. Furthermore, we perform a quantitative assessment of our tool using the gold standard annotations from human prefrontal cortex and hippocampus trisynaptic spatial datasets^32^. To further test the robustness of SpatialPrompt, we perform spot deconvolution on the mouse kidney spatial dataset using two publicly available unpaired scRNA-seq reference datasets^33,34^. We also built an open-access scRNA-seq database having more than 40 manually annotated reference datasets to facilitate seamless integration during spatial deconvolution using SpatialPrompt.

## Results

### Overview of SpatialPrompt

**Fig. 1:**
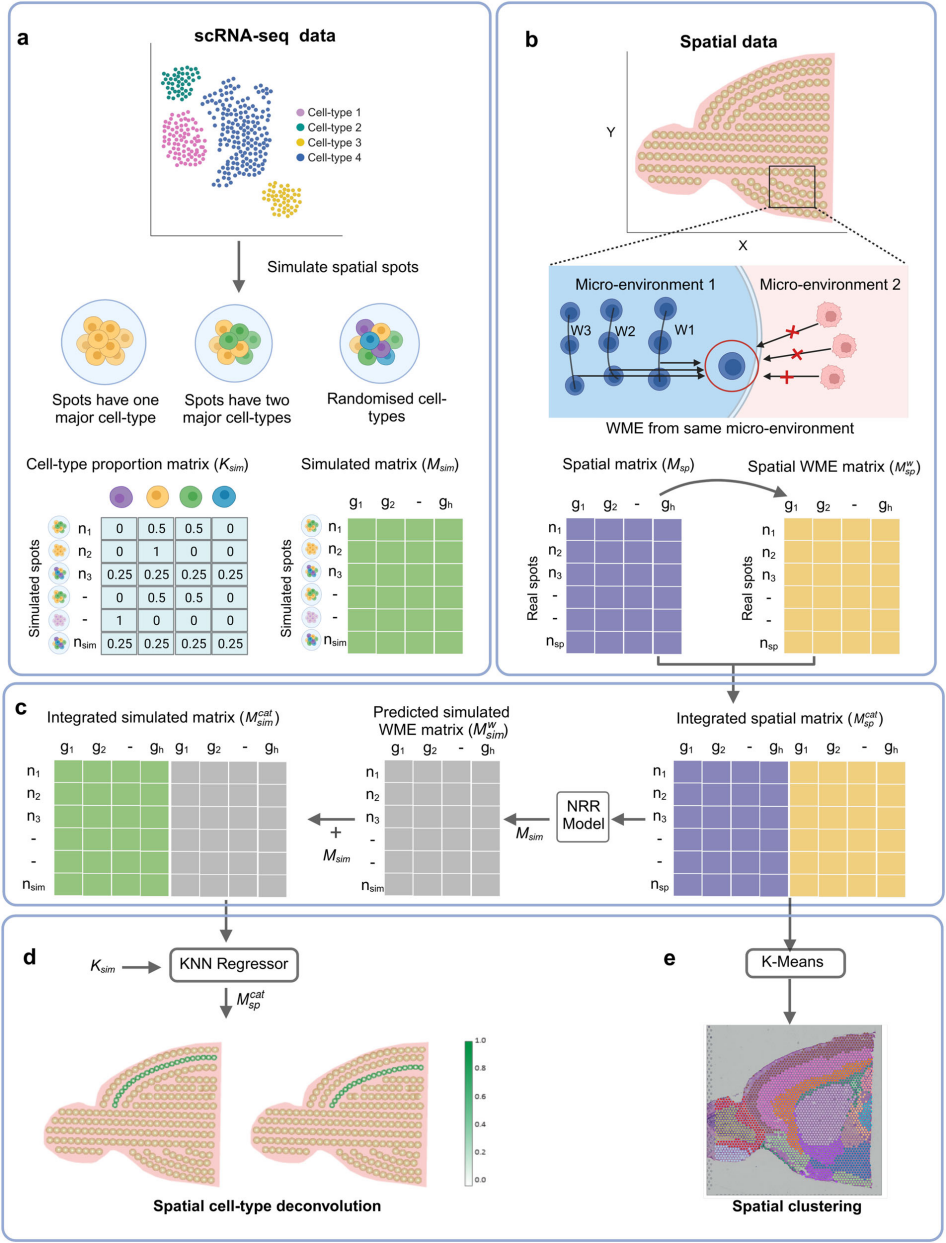
Overall workflow of SpatialPrompt. SpatialPrompt framework takes as input the spatial matrix (*M*_*sp*_) with spatial coordinate information and single-cell RNA-seq (scRNA-seq) matrix (*M*_*sc*_) with cell-type annotations. **a** The custom spatial spot simulator utilises *M*_*sc*_ and cell type annotations to generate simulated expression matrix (*M*_*sim*_) with known cell type proportion matrix (*K*_*sim*_). Spatial data is simulated under three scenarios to mimic the real spatial data, **b** *M*_*sp*_ and spatial coordinates is used to calculate the weighted mean expression (WME) from neighbours in same micro-environment for each spatial spot in *M*_*sp*_ the matrix ${M}_{{sp}}^{w}$ stores the WME value for *n*_*sp*_ real spatial spots, **c** The non-negative ridge regression (NRR) model is built using integrated spatial matrix ${M}_{{sp}}^{{cat}}$. Next, the NRR model is employed to predict the WME for each simulated spot in *M*_*sim*_ by utilising real spatial expression in *M*_*sp*_ and its weighted mean neighbour expression in ${M}_{{sp}}^{w}$. The integrated simulated matrix ${M}_{{sim}}^{{cat}}$ is obtained by combining *M*_*sim*_ and ${M}_{{sim}}^{w}$ column-wise, **d** For spatial deconvolution, KNN regressor model is trained on (${M}_{{sim}}^{{cat}}$) and *K*_*sim*_. Then, this model predicts the cell type proportions in real spatial matrix (${M}_{{sp}}^{{cat}}$), **e** For domain identification, spatial clustering is performed using K-means algorithm on the integrated spatial matrix (${M}_{{sp}}^{{cat}}$). This figure created with BioRender.Com.

SpatialPrompt framework learns the cell-type specific gene signatures from the scRNA-seq reference dataset and performs spatially informed spot deconvolution. The framework of SpatialPrompt operates in a stepwise manner. In step 1, using the reference scRNA-seq dataset (i.e., *M*_*sc*_ matrix), our spatial simulator simulates spatial spots (*M*_*sim*_) under three different scenarios to mimic the real spatial data (Fig. 1a). The basic idea behind the spatial simulator is that spots residing in the core of the microenvironment predominantly consist of one cell type and spots reside at the border of microenvironments have a mixture of cell types. In step 2, an iterative approach inspired by the message-passing layer in the graph neural net was applied to incorporate the local microenvironment information (${M}_{{sp}}^{w}$) into the spatial data (Fig. 1b). This step calculates the weighted mean expression (WME) of the local microenvironment for each spatial spot. Next, a concatenated representation of the spatial data $({M}_{{sp}}^{{cat}})$ was created by combining the spot’s own expression (*M*_*sp*_) and local WME (${M}_{{sp}}^{w}$). Thus, ${M}_{{sp}}^{{cat}}$ captures the spot’s individual characteristics along with the local microenvironment information. In step 3, a NRR model was fit to the matrix ${M}_{{sp}}^{{cat}}$ to learn the local microenvironment gene expression patterns from the real spatial data (Fig. 1c). This model was employed to predict the spatial pattern $({M}_{{sim}}^{w})$ in the simulated spatial data (*M*_*sim*_) (see above, step 1). Lastly, in step 4, KNN regressor model is used for spot deconvolution (Fig. 1d). This model learns the cell type specific gene signatures from the spatial embedded simulated matrix and predicts the cell type proportions in the real spatial data. Further, the k-means clustering is applied on the concatenate matrix, ${M}_{{sp}}^{{cat}}$, for domain identification (Fig. 1e).

### Benchmarking analysis on human DLPFC and hippocampus Slide-seq datasets

**Fig. 2:**
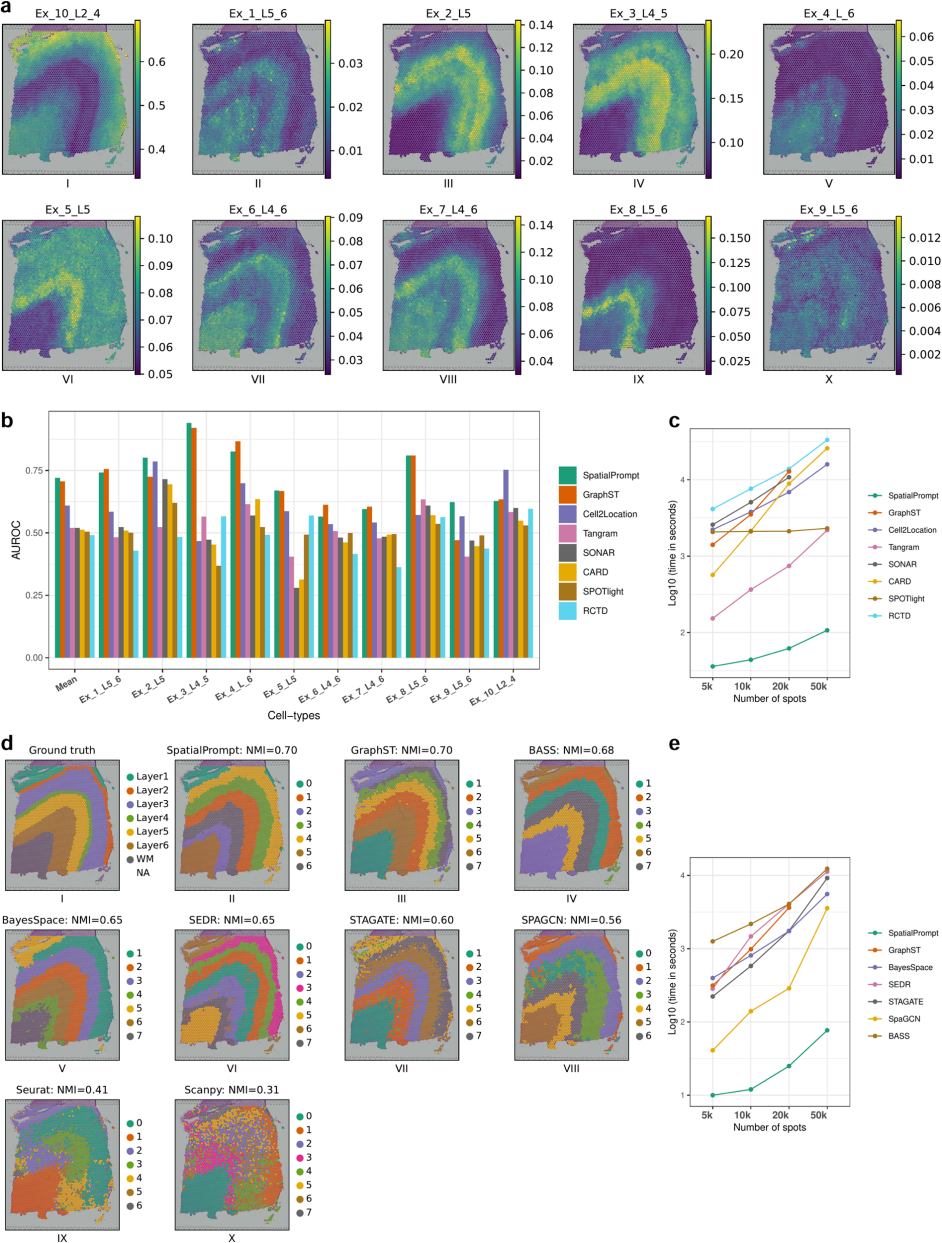
Quantitative assessments of various tools for spot deconvolution and domain identification using the human dorsal prefrontal cortex (DLPFC) and mouse hippocampus datasets. **a** Cell-type distribution of ten major neuronal cell types predicted by SpatialPrompt on human DLPFC dataset. **b** Bar plot of area under receiver operating characteristic curve (AUROC) scores obtained by comparing gold standard annotations of seven layers in human cortex. Higher AUROC scores indicate better accuracy and performance. **c** Comparison of runtime of all tools for varying number of spatial spots obtained from mouse hippocampus dataset. The horizontal and vertical axes represent the number of spatial spots and the logarithmic scale of the running time (in seconds) for each tool, respectively. **d** Spatial clustering performance of all domain identification tools on human DLPFC dataset. The NMI score is used to measure the similarity between the ground truth (sub-panel I) and the clusters predicted by each method (sub-panels II to X). Higher NMI scores indicate better clustering accuracy. **e** Comparison of runtime of SpatialPrompt and all spatial-based clustering tools for varying number of spatial spots obtained from mouse hippocampus dataset. The horizontal and vertical axes represent the number of spatial spots and the logarithmic scale of the running time (in seconds) for each tool, respectively. GraphST and SONAR were unable to perform on the spatial dataset having 50,000 spots due to high memory usage.

The performance of SpatialPrompt and seven existing spot deconvolution methods (CARD^10^, Cell2location^12^, Tangram^35^, SPOTlight^9^, GraphST^14^, SONAR^15^, and RCTD^11^) was evaluated using the human DLPFC spatial dataset. At first, spatial data and their corresponding reference scRNA-seq datasets were integrated. Then, all the methods were applied on this integrated dataset to calculate the cell-type proportions in the spatial spots. For all methods, the layer discriminative accuracy, based upon predicted cell-type proportions and ground truth layer annotation, was assessed by calculating the area under receiver operating characteristics (AUROC) score. Our benchmarking analysis showed that SpatialPrompt was able to capture the cell-type topography for all ten excitatory neurons (Fig. 2a).

Noteworthy, SpatialPrompt showed superior performance as compared to all seven methods with a mean AUROC score of 0.72 (Fig. 2b). GraphST was the second-best method next to SpatialPrompt with a mean AUROC score of 0.70. The mean AUROC scores for the remaining methods were found to be alike with Cell2location 0.60, Tangram 0.52, SONAR 0.52, CARD 0.51, Spotlight 0.50, and RCTD 0.49 (Supplementary Figs. 1–6).

The total running time of all methods was calculated on the hippocampus Slide-seq dataset comprising 53,173 spots and 23,264 genes. At first, this spatial dataset was used to create four low-resolution spatial subsets with varying number of spots as described in the method section. Our results show that SpatialPrompt exhibited superior performance with upto 20 times faster as compared to the other benchmarked methods (Fig. 2c and Table 1). Moreover, SpatialPrompt showed high scalability by efficiently handling thousands of spatial spots. Indeed, SpatialPrompt is the only method capable of performing spatial deconvolution on a dataset comprising 50,000 spots in under 2 min. Except Tangram, other methods (CARD^10^, Cell2location, SPOTlight^9^, GraphST^14^, SONAR^15^, and RCTD^11^) exhibited runtime of over 35 min to handle even a smaller spatial dataset containing only 10,000 spatial spots (Fig. 2c). The RCTD tool performed poorly among compared methods with runtime more than 2 hours (Table 1).

**Table 1:** Shows comparative quantitative assessment of benchmarked tools for spot deconvolution using human dorsal prefrontal cortex (DLPFC) and Slide-seq datasets

Tools	PMID	AUROC	Time (in seconds)			
			T1	T2	T3	T4
SpatialPrompt	-	0.72	36	44	62	107
GraphST	36859400	0.70	1407	3520	12859	NA
Cell2location	35027729	0.61	2225	3793	6894	15915
CARD	35501392	0.51	568	2139	8892	25797
Tangram	34711971	0.52	153	365	745	2189
SONAR	37550279	0.52	2580	5076	10800	NA
SPOTlight	33544846	0.51	2073	2109	2119	2310
RCTD	33603203	0.49	4140	7632	13896	33192

*Tools*: name of tools used for benchmarking deconvolution performance; *PMID*: PubMed identifier referring to research paper of the respective tools; dash (-) indicated this tool was not available on PubMed; *AUROC*: area under the receiver operating characteristics scores obtained by comparing the tool’s output to the ground truth annotation in DLPFC dataset; *Time*: runtime (in seconds) to process 5000 spots (T1), 10,000 spots (T2), 20,000 spots (T3), and 50,000 (T4) spots obtained from Slide-seq dataset. NA: GraphST and SONAR were unable to perform spot deconvolution on the spatial dataset having 50,000 spots due to high memory usage.

Similarly, the domain identification performance was assessed using slide “151673” of the human DLPFC dataset. For this benchmarking analysis, we selected six spatial-based (GraphST^14^, BASS^25^, BayesSpace^21^, SEDR^24^, STAGATE^23^, and SPAGCN^22^) and two non-spatial based (Seurat^19,36^ and Scanpy^20^) methods. SpatialPrompt, and GraphST both achieved the highest normalised mutual information (NMI) score of 0.70, indicating their superior performance compared to other tools (Fig. 2d and Table 2). Among the other spatial-based tools, BASS, BayesSpace and SEDR also performed well with an NMI score of ≥0.65. The remaining two methods had NMI score ≤0.60. In contrast, non-spatial methods *viz. Seurat* and *Scanpy* performed poorly with NMI scores 0.41 and 0.31, respectively. Furthermore, our runtime analysis using the hippocampus Slide-seq dataset revealed that all spatial-based methods scaled poorly for domain identification when the number of spots increased >5000 (Fig. 2e). In contrast, SpatialPrompt could successfully identify domains in the spatial dataset with 50,000 spots within 90 s. Though GraphST and BASS performed well compared to other tools, in terms of scalability, they were not able to scale when the number of spots is >10,000 (Fig. 2c, e). Overall, our analysis shows that SpatialPrompt was 44–150 times faster compared to other domain identification tools.

**Table 2:** Shows the quantitative assessment result for domain identification using human dorsal prefrontal cortex (DLPFC) and Slide-seq dataset

Tools	PMID	NMI	Time (in seconds)			
			T1	T2	T3	T4
SpatialPrompt	-	0.70	10	12	25	77
GraphST	36859400	0.70	314	984	3642	NA
BASS	35927760	0.68	1260	2178	4068	12369
STAGATE	35365632	0.60	223	581	1752	9215
SEDR	38217035	0.65	284	1471	4102	11264
SpaGCN	34711970	0.56	41	140	288	3572
BayesSpace	34083791	0.65	398	809	1740	5580

*Tools*: domain identification tools included in benchmarking spatial clustering performance; *PMID*: PubMed identifier referring to research paper of the respective tools; dash (-) indicated this tool was not available on PubMed or unpublished; *NMI*: normalised mutual information scores obtained by comparing the tool’s output with the ground truth annotation in DLPFC dataset; *Time*: runtime (in seconds) to process 5000 spots (T1), 10,000 spots (T2), 20,000 spots (T3), and 50,000 (T4) spots obtained from Slide-seq dataset. NA: GraphST was unable to identify domains on the spatial dataset having 50,000 spots due to high memory usage.

### Application of SpatialPrompt on mouse brain Visium and Slide-seq datasets

We assessed the robustness of SpatialPrompt using the mouse cortex Visium and hippocampus Slide-seq datasets. At first, these two datasets were pre-processed as described in the method section. Next, we applied SpatialPrompt, GraphST, and Cell2location tools separately on both datasets for spot deconvolution. Here, GraphST and Cell2location were chosen as they were the best-performing methods after SpatialPrompt in the previous benchmarking analysis.

**Fig. 3:**
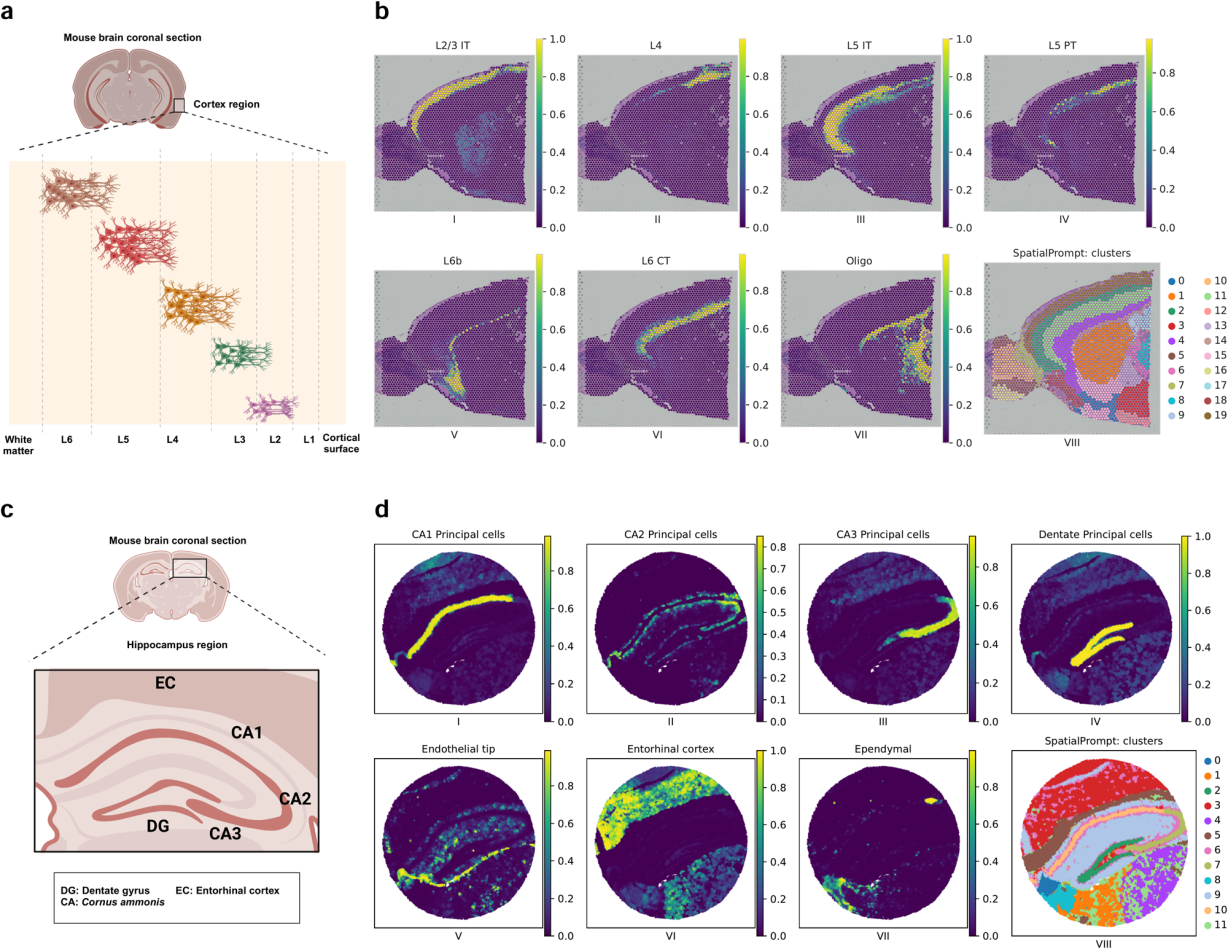
Spatial mapping of major cell types on mouse cortex and hippocampus. **a** Graphic illustration of sequential organisation of major cell types in mouse cortex from layers L1 to L6 and white matter. **b** Spatial mapping of seven major cell types in mouse Visium cortex by SpatialPrompt. This Visium spatial dataset used here consists of 2559 spots. The sub-panel VIII (bottom right) shows the spatial domains obtained by SpatialPrompt. **c** Graphic illustration of organisation of major cell types in mouse hippocampus region. **d** Spatial mapping of major cell types in hippocampus region by SpatialPrompt. Here, the Slide-seq dataset used comprised 53,173 spots. The sub-panel VIII (bottom right) shows the domains obtained by SpatialPrompt. Figure panels 3a and 3c were created with BioRender.Com.

The excitatory neurons in the mouse cortex are arranged sequentially in six layers labelled as L1 to L6 (Fig. 3a). Interestingly, each layer has specific gene signatures and molecular function^37^. The layers L1 to L3 are rich in synaptic connections that facilitate intracortical communication^38^. The layer L4 is the main receiver of inputs coming from the thalamus^39^. The layers L5 and L6 are the cortex deep layers that have major role in long-distance projections to thalamus, striatum, and spinal cord^40^. Noteworthy, SpatialPrompt was able to predict each cell type proportions in the Visium dataset with more clear sequential organisation as compared to Cell2location and GraphST (Fig. 3b and Supplementary Figs. 7 and 8). A recent study reported that most deconvolution tools applied to the same Visium dataset falsely predicted the L2/3 cell type in the centre of the cortex region (caudoputamen and nucleus accumbens area)^41^. Likewise, we noticed that both Cell2location and GraphST also made an incorrect prediction on L2/3 cell type in the centre cortical region (Supplementary Figs. 7 and 8). In contrast, SpatialPrompt predicted almost negligible presence of L2/3 cells in the cortical centre region (Fig. 3b).

Hippocampus majorly comprises three regions viz. cornu ammonis 1-2 (CA1-2), cornu ammonis 3 (CA3), and denate gyrus (DG), as shown in Fig. 3c^10,42^. The CA1 region is positioned closest to the subiculum, while CA3 is positioned adjacent to the DG. The CA2 is situated between CA1 and CA3. The DG has a ‘denate’ or tooth-like appearance near CA3^43^. Entorhinal cortex (EC) lies in the medial temporal lobe, which is the main interface between the hippocampus and neocortex^44^. We noticed that SpatialPrompt precisely predicted the cell types of all anatomical features especially for CA1, CA2, CA3, DG, and EC (Fig. 3d). In contrast, Cell2location was able to capture only a few of the anatomical features of certain cell types, such as CA3 and ependymal cells (Supplementary Fig. 9). Despite a runtime of 4.4 h, the Cell2location tool incorrectly predicted some proportion of CA1 cells in the EC region and vice-versa. Furthermore, it predicted denate cells in CA1, CA2, and entorhinal region. Conversely, SpatialPrompt achieved optimal predictions with an impressively shorter runtime of 2 min.

Similarly, we applied SpatialPrompt for domain identification on both datasets. On the Visium cortex dataset, clusters labelled as 5, 2, 7, 4, and 14 were aligned sequentially, resembling the organisation of cortical layers L2/3 to L6 (Fig. 3b, sub-panel VIII). Likewise, on the Slide-seq dataset, precise clusters were obtained revealing the distinct anatomical features in the hippocampus (Fig. 3d, sub-panel VIII). Specifically, cluster 10 predominantly consisted of CA1 and CA2 cell types, while cluster 7 majorly comprised CA3 cell types. Furthermore, cluster 2 was associated with DG cell type and cluster 3 had EC cell type. As this dataset was comprised of 53,173 spots, GraphST was unable to deconvolute and cluster the spatial spots due to high memory usage.

### Benchmark SpatialPrompt performance on hippocampus Slide-seq trisynaptic dataset

**Fig. 4:**
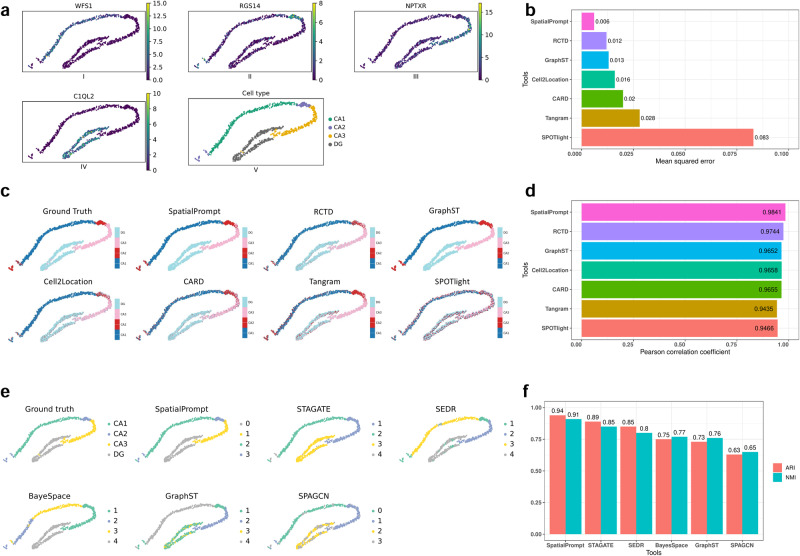
Quantitative assessment of all tools on hippocampus Slide-seq trisynaptic dataset. **a** Expression levels of *WFS1, RGS14, NPTXR*, and *C1QL2*, which are the marker genes for CA1, CA2, CA3, and DG cell type, respectively. **b**, **d** Bar plot of mean squared error and Pearson’s correlation coefficient obtained by comparing the cell type proportions predicted by the tools and the ground truth. **c** Spot deconvolution plots produced by all the tools; each colour represents a cell type. **e** Spatial clustering performance of all spatial-based domain identification tools, each colour represents a domain. **f** Bar plot of NMI and ARI scores obtained by comparing the ground truth annotations and the predicted annotations by the tools, higher ARI and NMI scores represent better clustering performance.

To further quantify the prediction accuracy of SpatialPrompt with other methods, the mouse hippocampal trisynaptic circuit Slide-seq dataset was subsampled. To subsample the dataset from the whole hippocampal Slide-seq data, domains with higher expression of *WFS1, RGS14, NPTXR*, and *C1QL2* were selected (Fig. 4a). Next, cells with high expression levels of these markers were manually annotated as CA1, CA2, CA3, and DG (Fig. 4a, sub-panel V). These cell type-specific markers were reported in the earlier studies^10,45^. As the Slide-seq dataset already has near-single-cell resolution, multi-cellular spots were created with known cell-type proportions (details in the method section). Next, the performance of all tools was evaluated using this dataset.

In terms of spot deconvolution, SpatialPrompt outperformed other tools with low mean squared error (MSE) and the highest Pearson’s correlation coefficient (PCC) of 0.005 and 0.98, respectively (Fig. 4b, d). RCTD, GraphST, SONAR, Cell2location, and CARD also performed well having PCC > 0.95 (Fig. 4d). Moreover, SpatialPrompt and RCTD both were able to distinguish the cell types with progressive edges compared to other methods (Fig. 4c). BayesTME does not require scRNA-seq reference for spot deconvolution; it predicts cell type profiles using an unsupervised approach. From the predicted cell types, none of the cell type profiles show association with any ground truth cell types (Fig. 4c), so MSE and PCC were not calculated for BayesTME predictions. For domain identification, SpatialPrompt was able to capture all four domains with the highest NMI and ARI (adjusted rand score) scores of 0.94 and 0.91, respectively (Fig. 4f). STAGATE and SEDR were also able to identify all four domains with less noise (Fig. 4e).

### Application on spatial mouse kidney dataset

**Fig. 5:**
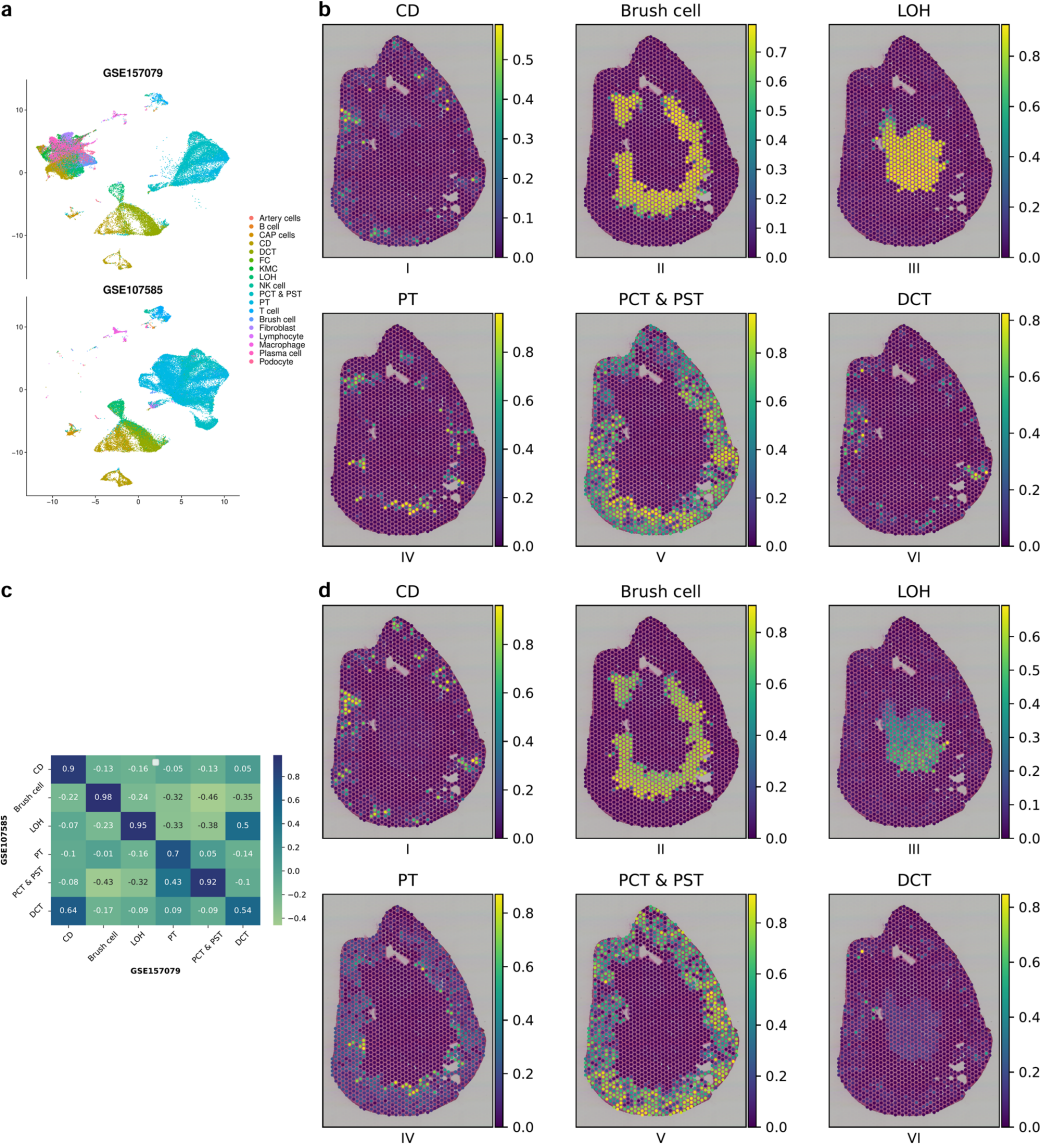
Cell type inference by SpatialPrompt in mouse kidney dataset using two different scRNA-seq references. **a** UMAP plot of GSE157079 (top) and GSE107585 (bottom) scRNA-seq reference datasets showing 18 cell-type clusters. **b**, **d** Spatial distribution of six major cell types predicted by SpatialPrompt in mouse kidney dataset using GSE157079 and GSE107585 as reference, respectively. **c** Heatmap of Pearson’s correlation coefficient calculated between the spot deconvolution predictions for six major cell types obtained using GSE157079 and GSE107585 as reference. CD collecting ducts, LOH loop of Henle, PT proximal tubule, PCT proximal convoluted tubule, PST proximal straight tubule, DCT distal convoluted tubule.

The performance of spot deconvolution methods varies remarkably depending on the choice of scRNA-seq reference^46,47^. To minimise this platform and batch effect arising due to different scRNA-seq references, SpatialPrompt employed several strategies. First, the tool employed counts per million normalisations applied to both reference and spatial datasets. Second, a non-negative ridge regression (NRR) model was employed that transfers the spatial microenvironment relation from the real spatial data to the simulated spatial data created from the scRNA-seq dataset. To assess the robustness of SpatialPrompt, we used the mouse kidney spatial dataset and two unpaired scRNA-seq reference datasets with accession id GSE157079 and GSE107585. The UMAP plot shows the distribution of different cell types in the two scRNA-seq datasets (Fig. 5a).

Our analysis shows that SpatialPrompt was able to capture the major cell-types distribution in the kidney spatial dataset using two different references separately (Fig. 5b, d). The major cell types, including collecting ducts (CD), proximal convoluted tubule (PCT), and proximal straight tubule (PST) were observed in the outer cortex region (Fig. 5b, d). Additionally, brush cells and loop of Henle (LOH) cell types extend from the outer cortex into the inner medulla region. Figure 5c shows the heatmap of Pearson’s correlation coefficient (*r*^2^) calculated between the spot deconvolution predictions for six major cell-types obtained using GSE157079 and GSE107585, respectively. We observed *r*^2^ score $\ge$ 0.9 for 4 cell-types, namely, collecting ducts, brush cell, loop of Henle, proximal convoluted tubule, and and proximal straight tubule cell-types. These results indicate an impressive robustness of SpatialPrompt while using different reference scRNA-seq datasets.

### Application of SpatialPrompt on MERFISH and STARmap spatial datasets

**Fig. 6:**
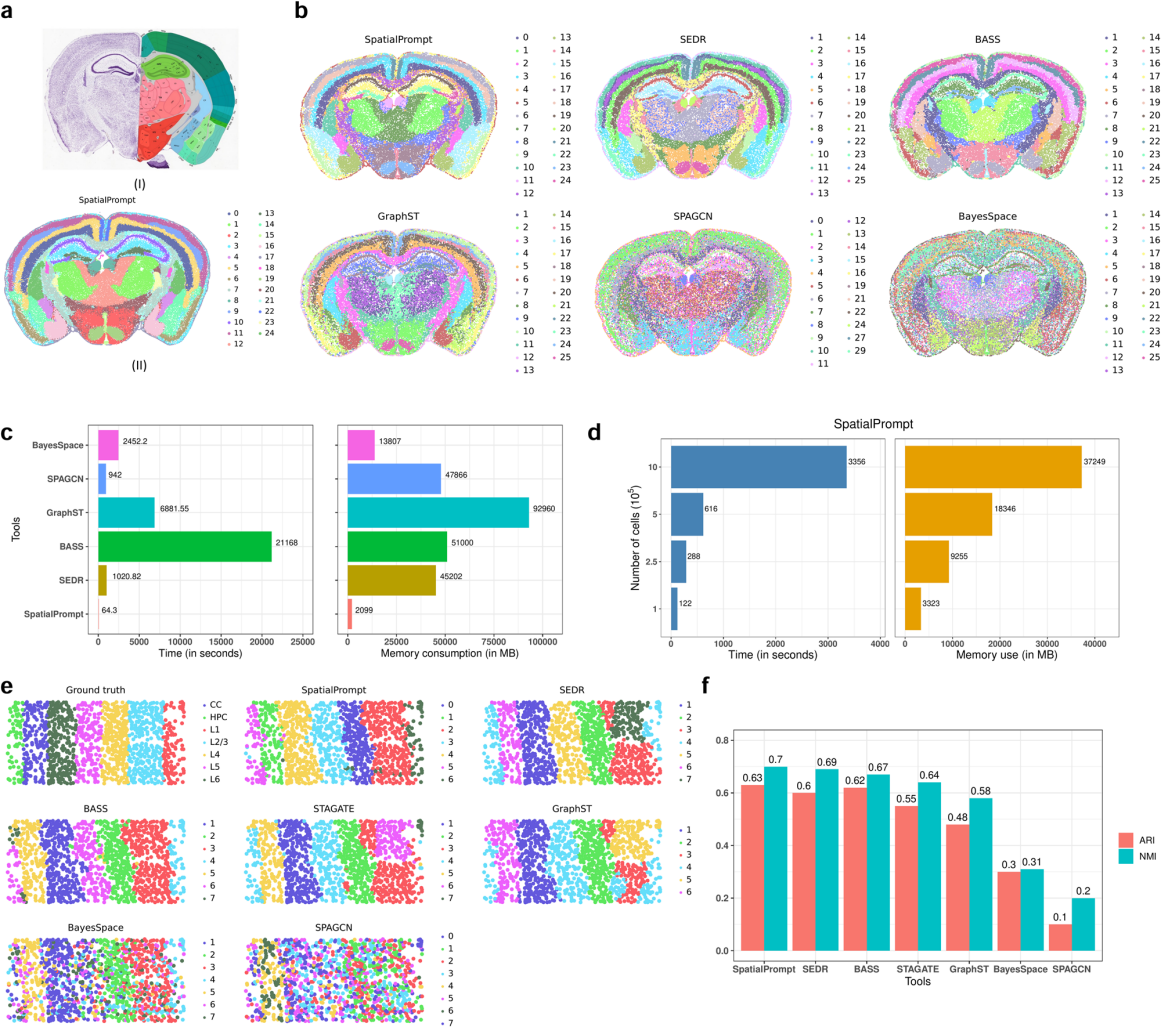
Evaluation of tools on MERFISH and STARmap datasets. **a** **I**. Major anatomical whole mouse brain features retrieved from the Allen Brain Atlas, **II**. Spatial domains captured by SpatialPrompt on the full MERFISH dataset having 1,19,927 cells. **b** Spatial clustering performance of domain identification tools on the sliced MERFISH data with 50,000 cells. **c** Comparison of all tools in terms of run-time and memory consumption on the sliced MERFISH data. **d** Run-time and memory consumption assessment of SpatialPrompt from ranging number of cells from 10^5^ to 10^6^. **e** Spatial clustering performance of all tools on the STARmap dataset. **f** Barplot of NMI and ARI scores obtained by comparing the ground truth annotations and predicted annotations by the tools.

We further evaluated the performance of SpatialPrompt and other existing tools on spatial datasets generated from in-situ hybridisation-based technologies. We used whole mouse brain MERFISH^30^ and mouse cortex STARmap^31^ datasets to perform the assessment. The MERFISH whole-brain spatial data was comprised of telencephalon, diencephalon, mesencephalon, and rhombencephalon. Each region has a spatial architecture. For instance, the telencephalon cerebral cortex has a sequential organisation from L1 to L6 celltype and hippocampal has the trisynaptic circuit of CA1, CA2, and CA3. Figure 6a (sub-panel I) shows a comprehensive view of mouse brain retrieved from Allen Brain Atlas^48^, where each colour represents one major domain. Similarly, in the STARmap mouse cortex spatial dataset, each colour represents the sequential organisation of L1 to L6 cell types.

Using MERFISH spatial dataset, SpatialPrompt successfully captured the major anatomical brain features with lesser noise compared to other existing tools (Fig. 6a, sub-panel II and Fig. 6b). Major brain anatomical features, like sequential cortical organisation, hippocampal trisynaptic circuit, pre-mammillary nucleus, lateral ventricle, and caudoputamen areas were clearly distinguishable by only SpatialPrompt, SEDR, and BASS (Fig. 6b). Among the remaining tools, GraphST exhibits lower noise in the detected domains compared to SPAGCN and BayesSpace. MERFISH datasets typically contain a large number (i.e., >50,000) of cells compared to sequencing-based spatial datasets (e.g., Visium). We assessed the computational efficiency of all tools in terms of both run-time and memory. For this purpose, a subset of MERFISH dataset having 50,000 cells was created (as described in the method section). We found that SpatialPrompt clearly outperform other tools in both execution time and memory usage (Fig. 6c). This shows SpatialPrompt is an ideal tool for large MERFISH datasets having millions of cells. Further, SEDR, BayesSpace, and SPAGCN were having moderate run-time and memory usage compared to other tools (Fig. 6c). GraphST, BASS and STAGATE exhibited poor performance. GraphST consumed nearly 92 GB of RAM and took ~1.91 h to execute. BASS used nearly 51 GB of RAM and took 5.88 h to process the dataset, while STAGATE required even larger RAM to process the MERFISH data having 50,000 cells (Fig. 6c). Further, scalability of SpatialPrompt was tested on MERFISH dataset by varying the number of cells from 10^5^ to 10^6^. Noteworthy, SpatialPrompt took less than 1 hour and 40 GB of memory to process 1 million cells (Fig. 6d). Conversely, some tools took more than 1 hour to process merely 50,000 cells.

Similarly, on STARmap dataset, SpatialPrompt performed well compared to other tools having highest NMI and ARI scores (Fig. 6e, f). SEDR and BASS also performed well, having NMI and ARI score of ≥0.60 and 0.69.

### Overview of SpatialPromptDB reference database

As SpatialPrompt tool can perform spot deconvolution using any scRNA-seq reference from the same tissue, we build SpatialPromptDB to provide compatible scRNA-seq references with cell-type annotations for seamless integration. Moreover, scRNA-seq datasets available at the GEO database, the human cell atlas, and the Broad Institute single cell portal were found to have inconsistent data formats. Importantly, most of these databases either do not provide cell type annotations or accessing them is quite complicated. To overcome these issues, we manually curated more than 40 scRNA-seq datasets with their cell-type annotations from different tissues for mouse and human. All scRNA-seq datasets were pre-processed as described in the method section. In addition, the cell type annotations provided by the respective studies were manually verified using the CellMarker database^49^. All the reference datasets can be downloaded by the user either from the SpatialPromptDB database or by using our python package (https://github.com/swainasish/SpatialPrompt).

## Discussion

Several computational tools have been developed for spot deconvolution and domain identification in spatial transcriptomics^9,12,21,22,35^. Majority of these existing tools ignore the valuable spatial information. Moreover, recently developed domain identification methods that utilise spatial coordinates run extremely slow for large datasets. To address these key issues, we introduced SpatialPrompt, a spatially aware, scalable, and accurate spot deconvolution and domain identification method for spatial transcriptomics.

Our benchmarking analysis clearly showed that SpatialPrompt outperforms the existing tools for both spot deconvolution and domain identification. For initial benchmarking study, we used the human DLPFC Visium dataset containing the gold standard manual annotation of 7 cortical layers^31^. Notably, SpatialPrompt achieved the highest AUROC score compared to all tools, and GraphST performed well with the DLPFC dataset. Next, we applied SpatialPrompt on two diverse publicly available mouse datasets to explore its robustness. The mouse cortex and hippocampus datasets used in our study were generated using low-resolution Visium and high-resolution Slide-seq techniques, respectively. We also applied GraphST and Cell2location on both datasets to draw comparisons. Interestingly, SpatialPrompt successfully captured the cell type organisation from L2/3 to L6 layers in the mouse cortex (Fig. 3b) with negligible false predictions compared to GraphST and Cell2location. Likewise, in the mouse hippocampus dataset, SpatialPrompt accurately captured the anatomical features of the major cell types. Next, a second benchmarking analysis was performed using the hippocampus Slide-seq trisynaptic circuit dataset. In both spot deconvolution and domain identification, SpatialPrompt was able to outperform other tools (Fig. 4). We also, extended our tool’s usability for domain identification to imaging-based spatial datasets, i.e., MERFISH and STARmap (Fig. 6). Domains identified by SpatialPrompt on both datasets exhibits lower noise compared to other tools.

The most important characteristic of SpatialPrompt is its scalability for larger datasets. We compared the runtime of all the methods included in our benchmarking analysis using the mouse hippocampus Slide-seq dataset with varying numbers of spatial spots (i.e., 5000 to 50,000). For a small dataset having 10,000 spots, the spot deconvolution using SpatialPrompt took 44 s, while other methods, except Tangram, took >35 min. Noteworthy, for a large dataset with 50,000 spots, SpatialPrompt took only 2 min, while other tools took hours to complete. Furthermore, domain identification using SpatialPrompt was 44–150 times faster compared to other methods. Clearly, SpatialPrompt is more accurate and highly scalable compared to the existing methods for spot deconvolution and domain identification. Imaging-based spatial datasets (e.g., MERFISH) usually have very high number of cells (>100k) compared to sequencing-based datasets, SpatialPrompt took less than 1 h and used less than 40 GB of memory to process 1 million cells. It’s worth mentioning that, in this scenario, other tools struggle to run when the number of cells exceeds 50,000.

Further, most of the existing tools provide inconsistent spot deconvolution prediction while using different reference scRNA-seq datasets^46,47^. We applied our SpatialPrompt tool on a kidney Visium dataset using two different scRNA-seq references. These two references were generated using different Illumina HiSeq platforms and at different geographic locations. Despite having technological and environmental differences in scRNA-seq references, SpatialPrompt was able to capture the major cell-type distribution in the kidney spatial data for both the scRNA-seq references (Fig. 4). Thus, SpatialPrompt provides consistent spot deconvolution for different scRNA-seq references. Moreover, we built SpatialPromptDB database that provides manually curated scRNA-seq reference datasets for seamless integration using SpatialPrompt tool. Our database harbours more than 40 scRNA-seq datasets from humans and mice with manually curated cell-type annotations that could be directly used in the SpatialPrompt tool. Hence, a researcher now does not need to perform separate scRNA-seq experiment for the purpose of spot deconvolution.

A few considerations are recommended for the better performance and accuracy of SpatialPrompt. Firstly, the scRNA-seq reference dataset to be used should capture diverse cell types and expression profiles, preferably from the same species. Also, wherever feasible, the cell-type annotations provided by the reference database should be used for spot deconvolution. Secondly, as a single scRNA-seq dataset may introduce potential bias to the results, users with multiple scRNA-seq reference datasets can address this by concatenating all references into one or running the tool independently for each reference. Third, the number of neighbours hyperparameter should be chosen carefully. For low-resolution 10X Visium^3^ and ST^5^ datasets, a value in the range 20–30 is recommended for this hyperparameter while a value in the range 50–60 is suggested for high-resolution Slide-seq^4^ datasets. For, very high-resolution datasets e.g., MERFISH, number of neighbours can be increase in the range of 100–150. However, users should be aware of a few limitations of the tool. One limitation involves choosing the hyperparameters. It is recommended to select the parameters suggested above or in the tutorials. Second, currently, our tool is able to identify domains in the two-dimensional space only, which can be extended to the three-dimensional in the future work. Third, it’s important to note that our tool is currently supports only a single reference input, can be extended to multiple references in the future work.

In summary, our comprehensive analyses using different spatial and scRNA-seq datasets demonstrated the robustness and superior performance of SpatialPrompt in inferring the spatial distributions of cell types and domains. Importantly, our analysis has shown that SpatialPrompt exhibits remarkable scalability. For instance, our tool can perform spot deconvolution and domain identification merely within 120 seconds even for a large dataset with 50,000 spots. Further, our findings revealed that SpatialPrompt performs consistently on different scRNA-seq reference datasets. Therefore, we also built SpatialPromptDB, a public database, that hosts more than 40 scRNA-seq datasets from humans and mice with manually curated cell-type annotations which could be directly used with our tool. We anticipate that SpatialPrompt will emerge as a preferred method by researchers targeting to perform spot deconvolution and domain detection using large-scale spatial datasets. Our tool is available open access as a Python package that can be easily integrated with Scanpy and similar popular pipelines.

## Materials and methods

### Datasets and pre-processing

We retrieved six spatial datasets and their corresponding scRNA-seq reference datasets from publicly available databases. All the spatial and scRNA-seq datasets were pre-processed using the *Scanpy* package^20^. Low-quality cells (or spots) and genes were removed from both spatial and scRNA-seq datasets. The details of all datasets (Supplementary Table 1) used for the purpose of this work are described below.

#### Human dorsal prefrontal cortex (DLPFC) dataset

This spatial dataset was collected from a database hosted at the Lieber Institute for Brain Development^32^. This dataset was generated through the sequencing of 12 tissue slides obtained from the human dorsal prefrontal cortex region. Following the previous studies^18,50,51^, the tissue slide numbered 151,673 was considered for the performance assessment of our tool. This chosen slide comprised 3639 spots and 33,538 genes. This spatial dataset has gold standard manual annotations of seven cortical layers (layer 1 to layer 6 and white matter), which is used as a ground truth for comparative evaluation of our method. The corresponding reference single-nucleus RNA sequencing (snRNA-seq) dataset comprising 78,886 cells and 30,062 genes was retrieved from the GEO database (accession id: GSE144136^52^).

#### Mouse 10X Visium cortex dataset

This spatial dataset was collected from the 10X Visium database, which was generated by sequencing the mouse cortex using Visium spatial technique^3^. This dataset has 2559 spots and 1337 genes from the anterior section. In the Visium spatial technique, mRNAs were captured from the beads having a diameter of 55 μm and bead-to-bead distance of 100 μm^3^. These low-resolution spots contain multiple cells (e.g., 10–50) depending on the cell type and cell class (e.g., normal or tumour). The corresponding scRNA-seq dataset of the mouse cortex was obtained from GEO database having accession ID GSE71585^53^. This scRNA-seq dataset has 14,249 cells and 34,617 genes.

#### Mouse hippocampus Slide-seq dataset

This spatial dataset was obtained from the mouse hippocampus, which has been sequenced using the Slide-seq technique^4^. Slide-seq is a high-resolution technique using a bead diameter of 10 μm, and each spot comprises 1–2 cells^4^. Both Slide-seq spatial dataset and corresponding reference scRNA-seq dataset were retrieved from the single cell portal^11^ hosted at the Broad Institute. This large spatial dataset has 53,173 spots and 23,264 genes. The sc-RNAseq dataset comprises 52,846 cells and 27,953 genes.

#### Mouse Visium kidney dataset

This spatial dataset was retrieved from mouse kidney and is available in the STOmicsDB^54^ database. In this dataset, five slides were sequenced at different time points. This dataset has 1617 spots and 32,285 genes. In addition, two mouse kidney scRNA-seq datasets were retrieved from GEO database having accession numbers GSE157079 and GSE107585. The GSE157079 dataset has 43,636 cells and 31,053 genes, and the GSE107585 dataset has 43,745 cells and 16,272 genes. The former was sequenced using the Illumina HiSeq 4000/3000 platform in the USA, while the latter was sequenced using the Illumina HiSeq 2000 platform in South Korea. Therefore, these two datasets entail diversity in their platform and environment.

#### Whole mouse brain MERFISH dataset

This spatial dataset of the whole mouse brain was retrieved from Allen Brain Atlas database^30,48^. Tissue section accession id: 638850 and its associated metadata were retrieved from the database. Retrieved dataset has 1,19,927 cells and 500 genes. In our benchmarking analysis, we noticed that many domain identification tools face challenges when processing spatial datasets with more than 50,000 cells (Fig. 2e). To assess their performance, we subsampled the MERFISH dataset from 119,927 cells to 50,000 cells. To benchmark memory and time consumption for the MERFISH whole brain dataset, four datasets were created having 100k, 250k, 500k, and 1 M cells, respectively. To generate the datasets, the required numbers of cells were randomly generated and indexed from the primary MERFISH dataset. Detail script about this simulation step was provided on the tool’s GitHub page.

#### STARmap mouse cortex dataset

This spatial dataset and cell type annotations of mouse cortex were retrieved from STAGATE paper. The dataset has 1207 cells and 1020 genes.

### Spatial spot simulation using reference scRNA-seq dataset

Instead of directly feeding the scRNA-seq data to the SpatialPrompt deconvolution module, we simulate the spatial spots from the reference scRNA-seq data using our custom simulator. Most of the existing spatial simulators have either one or more issues. For instance, many existing simulators simulate the spots by randomly merging the single cells^51^. In addition, these simulators often ignore the spots residing in the core of the microenvironment, which consist of one or two predominant cell types. As, spots reside in the core of microenvironment are less heterogenous compared to the spots reside in the border of microenvironments. For example, in the hippocampus, spots residing in the core of the CA1 region primarily comprise CA1 cells, while spots located in the transition from CA1 to CA2 regions, consist of a mixture of both celltypes. Our spatial simulator overcome these issues and simulate the spots following three scenarios. At first, the simulator generates spots that comprise of one major cell type, which resemble the spots that reside at the core of microenvironment or have less heterogeneity. Next, it simulates spots that predominantly consist of 2–3 cell types, which resemble the spots located at the border of two microenvironments or have moderate high heterogeneity. Lastly, the simulator randomly merges single cells to simulate spots that resemble the high heterogenous spots in the border of multiple microenvironments and rare cell types.

### SpatialPrompt for spatially informed spot deconvolution and clustering

The following notations are used to describe the framework of our SpatialPrompt tool:

*M*_*sc*_: scRNA-seq matrix having *n*_*sc*_ cells (rows) and *g*_*sc*_ number of genes (columns),

*M*_*sp*_: spatial RNA-seq matrix having *n*_*sp*_ spots (rows) and *g*_*sp*_ number of genes (columns),

*K*_*sc*_: column matrix storing cell-type annotations for the *n*_*sc*_ cells in *M*_*sc*_,

*M*_*x,y*_: stores x and y coordinates of the spots in *M*_*sp*_,

*g*_*c*_: number of common genes between *g*_*sc*_ and *g*_*sp*_,

*g*_*h*_: top high variance common genes (i.e., a subset of *g*_*c*_) identified from *M*_*sc*_,

${M}_{{sc}}^{h}$, ${M}_{{sp}}^{h}$: single cell and spatial matrices after indexing *g*_*h*_ genes from the *M*_*sc*_ and *M*_*sp*_, respectively,

${M}_{{sc}}^{n}$, ${M}_{{sp}}^{n}$: counts per million normalised single cell and spatial matrices, respectively,

${M}_{{sp}}^{w}$: local weighted mean expression of spatial spots in ${M}_{{sp}}^{n}$,

${M}_{{sp}}^{{cat}}$: column-wise concatenated matrix of ${M}_{{sp}}^{n}$ and ${M}_{{sp}}^{w}$ having dimension *n*_*sp*_*×* 2*g*_*h*_,

*M*_*sim*_: simulated spatial matrix having *n*_*sim*_ spots (rows) and *g*_*h*_ genes (columns),

*K*_*sim*_: known cell-type proportion matrix for the simulated spots in *M*_*sim*_,

${M}_{{sim}}^{w}$: predicted local weighted mean expression of *M*_*sim*_,

${M}_{{sim}}^{{cat}}$: column-wise concatenated matrix of *M*_*sim*_ and ${M}_{{sim}}^{w}$ having dimension *n*_*sim*_× 2*g*_*h*_,

*K*_*pred*_*:* predicted cell-type proportion matrix of the real spot matrix *M*_*sp*_.

Our SpatialPrompt tool requires *M*_*sc*_ and *M*_*sp*_ matrices along with *M*_*x,y*_ and *K*_*sc*_ for the spot deconvolution. For domain identification, SpatialPrompt need only *M*_*sp*_ and *M*_*x,y*_. At first, our tool identifies the common genes, *g*_*c*_, between *M*_*sc*_ and *M*_*sp*_. Of these *g*_*c*_ common genes, the *g*_*h*_ (default value is 1000) number of high-variance genes is obtained from *M*_*sc*_. After this, matrices ${M}_{{sc}}^{h}$ and ${M}_{{sp}}^{h}$ are created by indexing *g*_*h*_ genes from *M*_*sc*_ and *M*_*sp*_, respectively. Next, counts per million normalisation was applied as follows:

1 $${{M}}^{{n}}\left({normalised}\right)=\frac{{M}}{{\sum }_{{i}=1}^{{{n}}_{{s}}}{{M}}_{{i}}}* {10}^{6}$$

Here, *M* could be ${M}_{{sc}}^{h}$ (or ${M}_{{sp}}^{h}$) with *n*_*s*_ cells (or spots) and ${M}^{n}$ represent the normalised matrix ${M}_{{sc}}^{n}$ (or ${M}_{{sp}}^{n}$). Next, the ${M}_{{sc}}^{n}$ was used to simulate the *M*_*sim*_ matrix with known cell type proportions *K*_*sim*_ under three different scenarios as described above. By default, number of simulated spots, *n*_*sim*_ was set within the range of 20,000 to 30,000. To set the optimum value of *n*_*sim*_, benchmarking analysis was performed by simulating spatial spots in the range from 10^3^ to 10^6^ using the human DLPFC spatial dataset. We observed that SpatialPrompt performed consistently after *n*_*sim*_ reached 20,000. Therefore, the default value for *n*_*sim*_ was set between 20,000 and 30,000.

To incorporate the microenvironment information into the spatial matrix ${M}_{{sp}}^{n}$, the weighted mean expression was calculated for each spatial spot following a three-step process. In step 1, K nearest neighbours were identified for each spot using the Scipy *cKDTree* spatial module^55^. The *cKDTree* module identifies neighbours very fast by dividing the search space into smaller regions with hyperplanes and searching only the spots that are likely to contain the nearest neighbour^55,56^. Moreover, it avoids calculating distances to the points relatively far away from the query point, thus making its application ideal for large datasets. In step 2, for each spot *Sq*, the cosine similarity denoted as cos(*θ*) was calculated with its K neighbours as follows:

2 $$\cos \left(\theta \right)=\frac{{S}_{q}\cdot {S}_{k}}{{{{{{\rm{|}}}}}}{S}_{q}{{{{{\rm{||}}}}}}{S}_{k}{{{{{\rm{|}}}}}}}$$

Here, numerator denotes the dot product of *S*_*q*_ and *S*_*k*_ vectors, and $|{S}_{q}|$ and $|{S}_{k}|$ represents the magnitude (norm). We considered top 50% of the K highly similar neighbours for weighted mean expression calculation. In step 3, an iterative approach inspired from the message-passing layer in the graph neural net was applied to calculate the weighted mean expression matrix (${M}_{{sp}}^{w}$) for each spot as follows:

3 $${M}_{{sp}}^{w}=\mathop{\sum }_{i=0}^{p}\mathop{\sum }_{j=0}^{{n}_{{sp}}}{\bar{M}}_{{{sp}}_{j}}* {w}_{i}\left\{\begin{array}{c}{w}_{1}=1\\ {w}_{i}={w}_{1}/i\end{array}\right.$$

Here, ${\bar{M}}_{{{sp}}_{j}}$ is the mean expression of the neighbours of the *j*th spatial spot, $p$ is the number of iterations, message passing layer will incorporate weighted mean into the spot. *W*_*i*_ is the weight assigned to the mean expression in the *i*th iteration. In each iteration, *w*_*i*_ is reduced to *w*_*1*_*/i*, where *w*_*1*_ initialised as 1. This was done to ensure that higher weightage will be assigned to the nearer spots as compared to farther spots. It is recommended to set the value of parameter $p$ between 5 and 10, as after 5th iterations all the spots will have the weighted mean expression of their neighbours from the same microenvironment. Next, ${M}_{{sp}}^{n}$ and ${M}_{{sp}}^{w}$ matrices were concatenated column-wise to create the ${M}_{{sp}}^{{cat}}$. The matrix ${M}_{{sp}}^{{cat}}$ was further used for domain identification by applying k-means clustering algorithm.

### Non-negative ridge regression (NRR) model

The NRR model was built to predict the local weighted mean expression of the simulated spatial matrix. Thus, NRR model predicts the ${M}_{{sim}}^{w}$ matrix based on ${M}_{{sp}}^{w}$ and ${M}_{{sp}}^{n}$. The regression coefficient in the NRR model was obtained by minimising the below cost function:

4 $$min \left({\left|{M}_{{sp}}^{w}-\beta \cdot {M}_{{sp}}^{n}\right|}^{2}-\lambda {\left|\beta \right|}^{2}\right)$$

Here, *λ* is the penalty or alpha parameter and *β* denotes the regression coefficient of the NRR model. To determine the optimum *λ*, a subset of ${M}_{{sp}}^{w}$ and ${M}_{{sp}}^{n}$ was selected for the hyperparameter tuning. After obtaining the *β* on the optimised *λ*, ${M}_{{sim}}^{w}$ can be predict by,

5 $${M}_{{sim}}^{w}=\beta \times {M}_{{sim}}$$

In the next step, *M*_*sim*_ and ${M}_{{sim}}^{w}$ were concatenated to form the matrix ${M}_{{sim}}^{{cat}}$. Note that, now ${M}_{{sim}}^{{cat}}$ have the information of own expression and predicted weighted mean expression of all simulated spots. A KNN-regressor model^57^ was trained using the ${M}_{{sim}}^{{cat}}$ as feature matrix and *K*_*sim*_ as target. For each real spot in ${M}_{{sp}}^{{cat}}$, the KNN-regressor model finds the K most related simulated spots in the kernel space with least Minkowski distance. Subsequently, the model utilises this information to predict the cell type proportions matrix *K*_*pred*_ for the given real spot based on the characteristics of its K nearest neighbours in the simulated data.

### Benchmarking performance of SpatialPrompt on human DLPFC dataset

The gold standard annotations in the human DLPFC dataset were used to assess the performance of SpatialPrompt with previously existing methods for both spot deconvolution and domain identification. This spatial dataset was selected for performance assessment as it was also used for benchmarking by several previous studies^14,51,58^. For spot deconvolution, we compared our method with 8 different methods, namely, GraphST, CARD, Cell2location, Tangram, SONAR, SPOTlight, BayesTME, and RCTD. Among these chosen methods, CARD, Cell2location, and Tangram have been reported as the best-performing methods in a recent benchmarking study^59^. SPOTlight and RCTD are the most cited methods for spot deconvolution. GraphST, SONAR, and BayesTME^60^ were the most recent spatial-based tools, so added to the performance assessment. BayesTME performs spot deconvolution without using the scRNA-seq reference dataset, so only applied to the hippocampal trisynaptic circuit spatial dataset only, so that inferred cell types can be verified manually. We extracted layer specific 10 neuron cell-types from the reference snRNA-seq dataset corresponding to the human DLPFC region. The area under receiver operating characteristics (AUROC) score was computed to assess the overall performance of each method. In our case, AUROC analysis involved determining the sensitivity and specificity of a tool to classify a spot to a particular cell type.

Similarly, for benchmarking the domain identification performance of SpatialPrompt, we selected eight different tools *viz*. GraphST, BASS, BayesSpace, SEDR, STAGATE, SPAGCN, Scanpy, and Seurat. The accuracy of domain identification tools was evaluated by calculating the normalised mutual information (NMI) score. This score measures the effectiveness of a tool by calculating the mutual information between the predicted clusters and the gold standard annotations. The NMI score was calculated between predicted clusters (*Y*_*P*_) and ground truth annotations (*Y*_*R*_) using equation given below:

6 $${NMI}\left({Y}_{P},{Y}_{R}\right)=\frac{2* {MI}\left({Y}_{P},{Y}_{R}\right)}{H\left({Y}_{P}\right)+H\left({Y}_{R}\right)}$$

Here, ${MI}\left({Y}_{P},{Y}_{R}\right)$ term in the numerator denotes the mutual information between *Y*_*P*_ and *Y*_*R*_, while $H\left({Y}_{P}\right)$ and $H\left({Y}_{R}\right)$ represent the entropy of ${Y}_{P}$ and ${Y}_{R}$, respectively.

The time complexity was calculated using the hippocampus Slide-seq dataset. The same dataset was also chosen to assess the scalability of all the benchmarked methods, because it consists of some 53,173 spatial spots and 23,264 genes. From this Slide-seq dataset, 4 subsets of spatial datasets were randomly sliced containing 5000, 10,000, 20,000, and 50,000 spots. Since most of the previous spatial deconvolution tools were not designed for large reference datasets, the corresponding scRNA-seq reference dataset was down-sampled to 13,247 cells and 10,000 genes. During the down-sampling, the cell types having counts >1000 were trimmed down to 1000 by randomly selecting the cells and 10,000 high-variance genes were selected for the downstream analysis. We set the number of high-variance gene either as per the recommendation provided with each tool or between 1000 to 2000 in case the tool allowed. To benchmark memory and time consumption for the MERFISH whole brain dataset, 4 datasets were created having 100k, 250k, 500k, and 1 M cells, respectively. To generate the datasets, the required numbers cells were randomly generated and indexed from the primary MERFISH dataset as mentioned above. We followed the instructions available in the documentation of respective tools and used the default parameter unless mentioned explicitly. For *Cell2location* tool, the *max_epochs* parameter was set at 2000 (i.e., default value is 30,000) for 20,000 and 50,000 spots due to the heavy computational burden. Other parameters were set at their default values. For GraphST tool, default parameters applied according to the tutorial provided, in the last clustering step “mclust” parameter was selected without optional refinement. BayesTME does not require any scRNA-seq reference for spot deconvolution. Therefore, it was solely applied to the small hippocampal trisynaptic circuit spatial data to manually verify the cell proportions and compare them to the ground truth. BayesTME took ~3 h to deconvolute the spatial dataset containing 1007 spots, which is why it was not considered for further benchmarking to assess scalability. Clustering pipeline implemented in STAGATE, SEDR, BASS, and SpaGCN was followed from their respective GitHub repository with default parameters. All analyses were performed on a system with an Intel Xeon processor with 48 cores, 128 GB of RAM, and 4 GB of graphics memory.

### Generation of low-resolution hippocampal trisynaptic circuit spatial data

To fetch the spatial domain of hippocampal trisynaptic circuit area, cells with high expression of *WFS1, RGS14, NPTXR*, and *C1QL2* were selected and manually annotated as CA1, CA2, CA3, and DG. Given that the Slide-seq dataset has near-single-cell resolution, multi-cellular spots were generated by merging nearly 10–15 cells into one with known cell-type proportions. The performance of all 12 tools was then evaluated using this dataset. The accuracy of spot deconvolution results calculated using mean squared error (MSE) and Pearson’s correlation coefficient (PCC). MSE and PCC calculated between predicted cell type probability (Y_P_) and real cell type probability (Y_R_) by:

7 $${MSE}=\frac{1}{n}{\sum }_{i=1}^{n}{\left({Y}_{P}\left(i\right)-{Y}_{R}\left(i\right)\right)}^{2}$$

8 $${PCC}=\frac{{\sum }_{i=1}^{n}\left({Y}_{P}\left(i\right)-\overline{{Y}_{P}}\right)\left({Y}_{R}\left(i\right)-\overline{{Y}_{R}}\right)}{\sqrt{{\sum }_{i=1}^{n}{\left({Y}_{P}\left(i\right)-\overline{{Y}_{P}}\right)}^{2}{\sum }_{i=1}^{n}{\left({Y}_{R}\left(i\right)-\overline{{Y}_{R}}\right)}^{2}}}$$

The accuracy of domain identification results was calculated using NMI and ARI (adjusted rand score) by:

9 $${ARI}=\frac{{RI}-E\left[{{\mbox{RI}}}\right]}{max \left({RI}\right)-E\left[{{\mbox{RI}}}\right]}$$

Here, RI stands for rand index, it accesses the similarity between the predicted cluster labels and real cluster labels.

### SpatialPromptDB database

We created a SpatialPromtDB database using the Mkdocs client^61^ to store the scRNA-seq reference datasets along with their cell-type annotations. The scRNA-seq datasets of major tissue classes were obtained from different databases, including GEO^62^, human cell atlas^63^, single cell portal^64^ of Broad Institute and Figshare^65,66^. At first, all these downloaded scRNA-seq datasets were loaded and pre-processed using the *Scanpy* package. Cells with less than 500 reads and genes having total reads of less than 1000 were removed. Additionally, cells having >20% mitochondrial genes were excluded. Next, counts per million normalisation and logarithmic transformation were applied to each dataset. If the cell annotations were not provided by the original study, the clusters were obtained using the *pp.neighbours* function available in the *Scanpy*. Cell type or cluster-specific markers were identified using the *tl.rank_genes_groups* function. These markers were further validated manually using the CellMarker^49^ and PanglaoDB^67^ databases. To verify the cell type-specific markers, we selected the top 20 markers with the highest log fold change values and manually queried them in both databases. Next, the raw count matrix of the expression data and cell-type annotations were exported in tabular form from the *Scanpy*. Finally, all the reference datasets with their cell-type annotations were hosted on SpatialPromptDB database (URL: https://swainasish.github.io/SpatialPrompt).

### Statistics and reproducibility

All the scRNA-seq and spatial datasets used in the study are publicly available. Source data of the figures and source code of our tool are available on the GitHub repository (https://github.com/swainasish/SpatialPrompt) and Zenodo (10.5281/zenodo.11070217)^68^. A detailed documentation and tutorials of our tool and database are available at: https://swainasish.github.io/SpatialPrompt/. The code and scripts used for benchmarking analysis are available as Google colab notebook on the above-mentioned GitHub repository for the purpose of reproducibility.

## Acknowledgements

This project was supported by the Department of Biotechnology (project number BT/GenomeIndia/2018) and the Indian Institute of Technology, Jodhpur, India (project number I/SEED/PY/20200037).

### Supplementary information

Peer Review File

Supplementary Information

## Data Availability

Several scRNA-seq and spatial publicly available datasets were utilised in this study. The spatial human DLPFC dataset was retrieved from the LIBD database (https://research.libd.org/spatialLIBD/), and the corresponding snRNA-seq dataset was accessed from GEO (accession id: GSE144136). The spatial mouse Visium cortex dataset was retrieved from the 10X genomics database (https://www.10xgenomics.com/resources/datasets). The corresponding scRNA-seq accessed was collected from GEO (accession id: GSE71585) for our analysis. We used the raw processed data from https://satijalab.org/seurat/articles/spatial_vignette.html. The spatial Slide-seq dataset and the corresponding scRNA-seq retrieved from the Broad Institute single-cell portal (https://singlecell.broadinstitute.org/single_cell/study/SCP948/robust-decomposition-of-cell-type-mixtures-in-spatial-transcriptomics). The spatial mouse kidney data downloaded from STOmicsDB (https://db.cngb.org/stomics/datasets/STDS0000121) and two reference scRNA-seq data accessed from GEO (accession id: GSE157079, 107585). The MERFISH whole mouse dataset was retrieved from Allen Brain Atlas (https://allen-brain-cell-atlas.s3.us-west-2.amazonaws.com/index.html), with tissue slice accession id 638850. STARmap cortex spatial dataset was retrieved from STAGATE paper (https://stagate.readthedocs.io/en/latest/T9_STARmap.html). All numerical source data for graphs has been provided in the tool’s GitHub repository and also at Zenodo: (10.5281/zenodo.11070217).

## Code availability

SpatiaPrompt Python package and scripts used for benchmarking analysis are available on the GitHub: https://github.com/swainasish/SpatialPrompt and at Zenodo: (10.5281/zenodo.11070217). Detailed documentation and tutorials of our tool and database are available at: https://swainasish.github.io/SpatialPrompt/.
