# Supplementary material for: SpatialPrompt: spatially aware scalable and accurate tool for spot deconvolution and domain identification in spatial transcriptomics
Source: Commun Biol. 2024 May 25;7:639. doi: 10.1038/s42003-024-06349-5 (PMC11127982; doi:10.1038/s42003-024-06349-5)
Supplement: Supplementary file 2 — Supplementary Information [file 42003_2024_6349_MOESM2_ESM.pdf]

Supplementary Information

SpatialPrompt: spatially aware scalable and accurate tool for spot deconvolution and clustering in spatial transcriptomics

Asish Kumar Swain[1], Vrushali Pandit[1], Jyoti Sharma[1], and Pankaj Yadav[1,2*]

[1]Department of Bioscience & Bioengineering, Indian Institute of Technology, Jodhpur, Rajasthan, 342030, India

[2]School of Artificial Intelligence and Data Science, Indian Institute of Technology, Jodhpur, Rajasthan, 342030, India

[*]Corresponding author: pyadav@iitj.ac.in

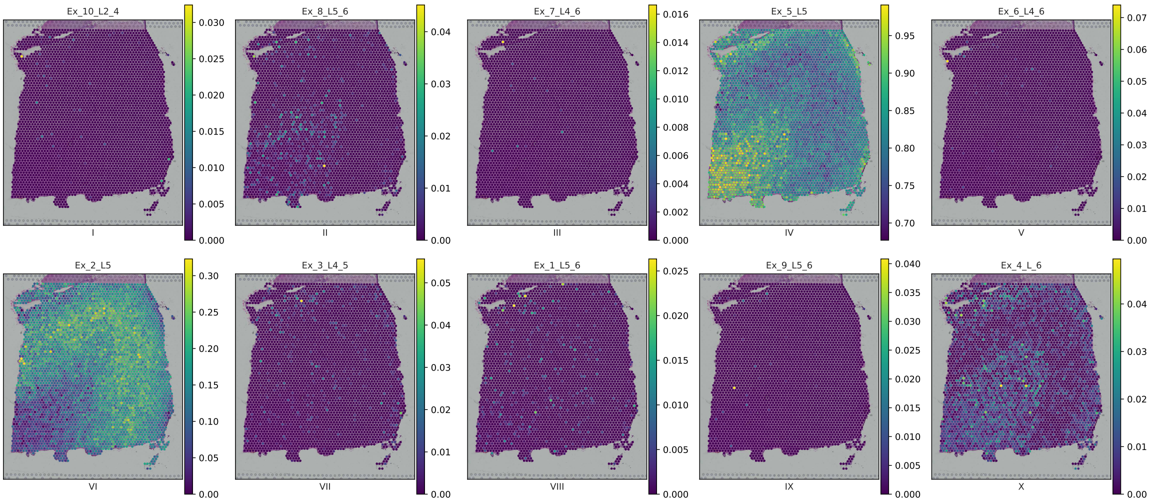

Supplementary Figure 1: Spatial mapping of major cell types in human DLPFC dataset by CARD tool.

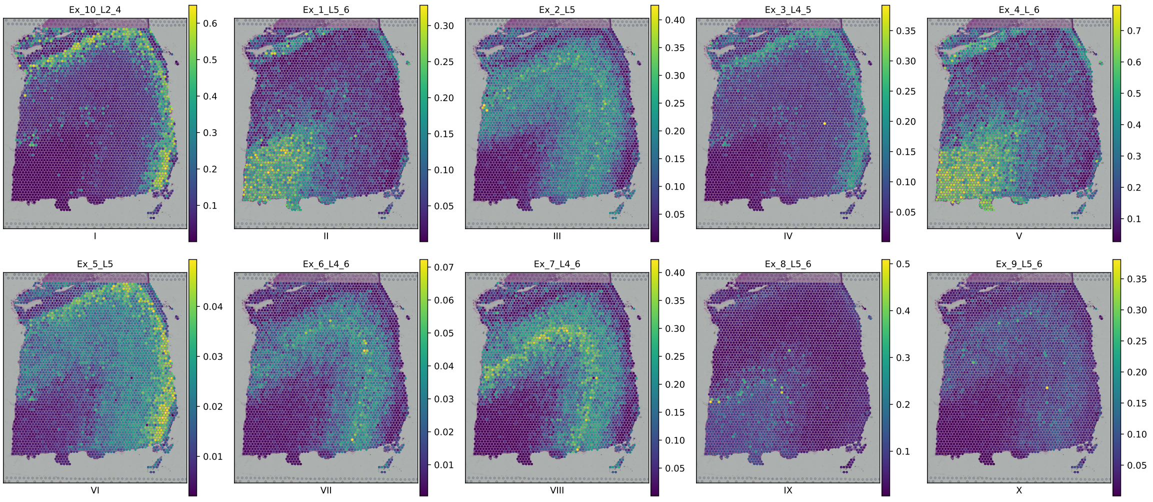

Supplementary Figure 2: Spatial mapping of major cell types in human DLPFC dataset by Cell2location tool.

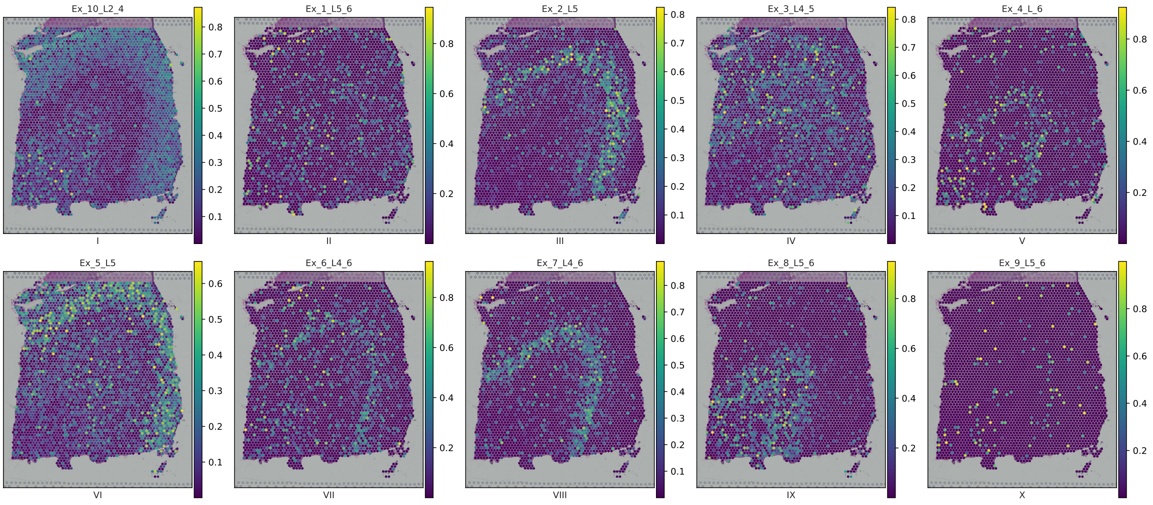

Supplementary Figure 3: Spatial mapping of major cell types in human DLPFC dataset by Tangram tool.

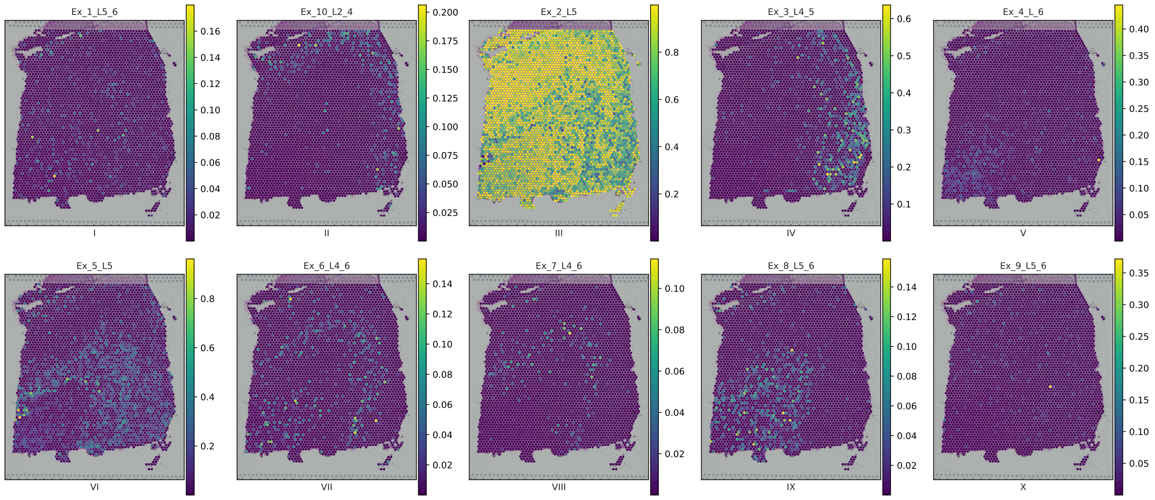

Supplementary Figure 4: Spatial mapping of major cell types in human DLPFC dataset by RCTD tool.

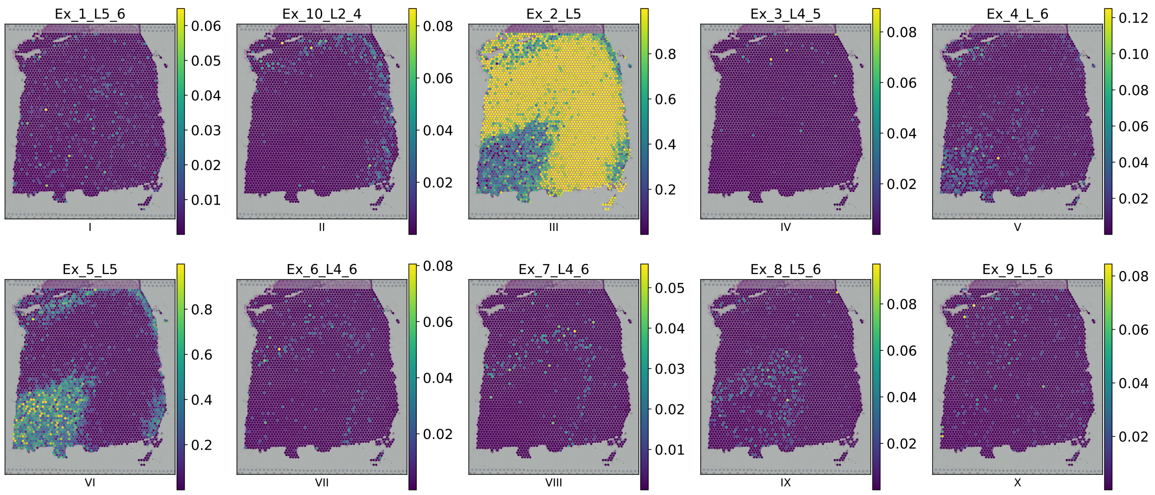

Supplementary Figure 5: Spatial mapping of major cell types in human DLPFC dataset by SONAR tool.

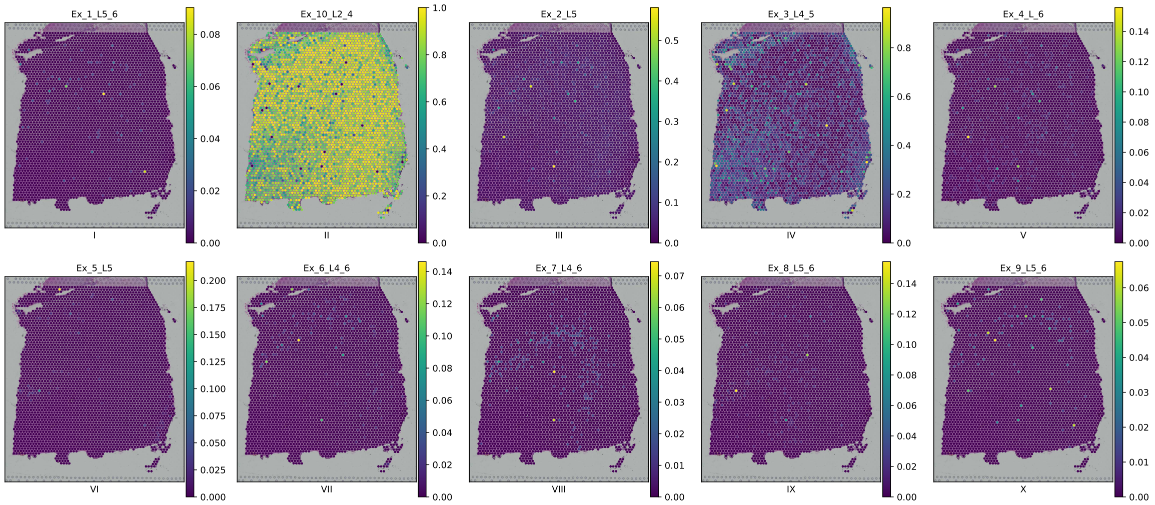

Supplementary Figure 6: Spatial mapping of major cell types in human DLPFC dataset by SPOTlight tool.

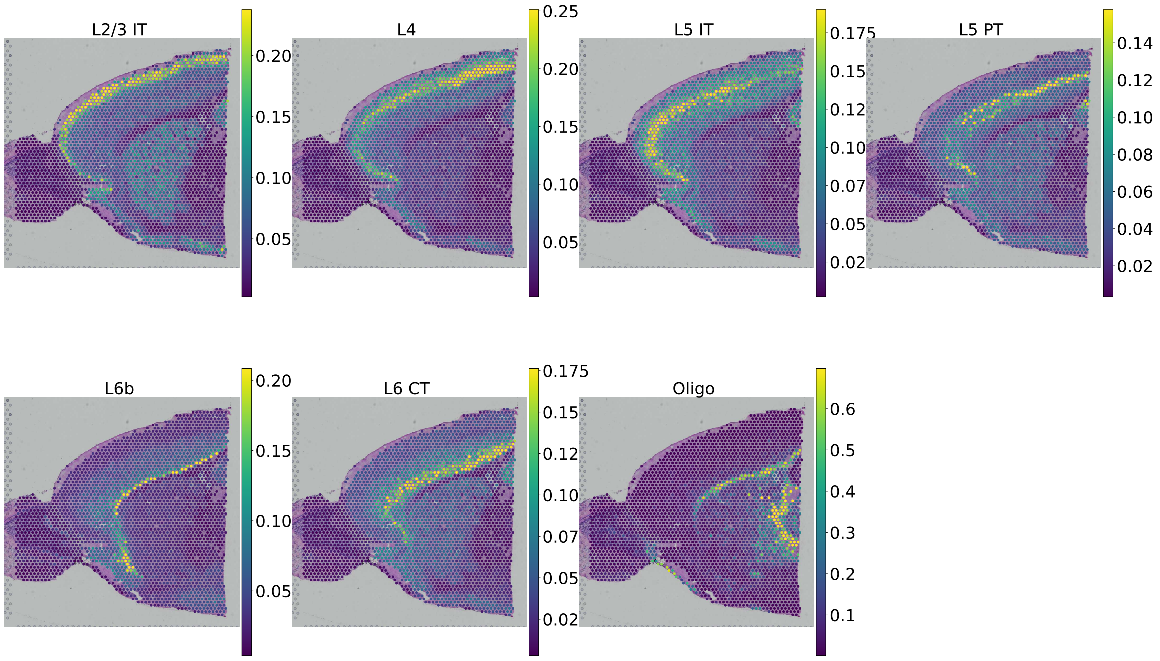

Supplementary Figure 7: Spatial mapping of major cell types in mouse Visium cortex dataset by Cell2location tool.

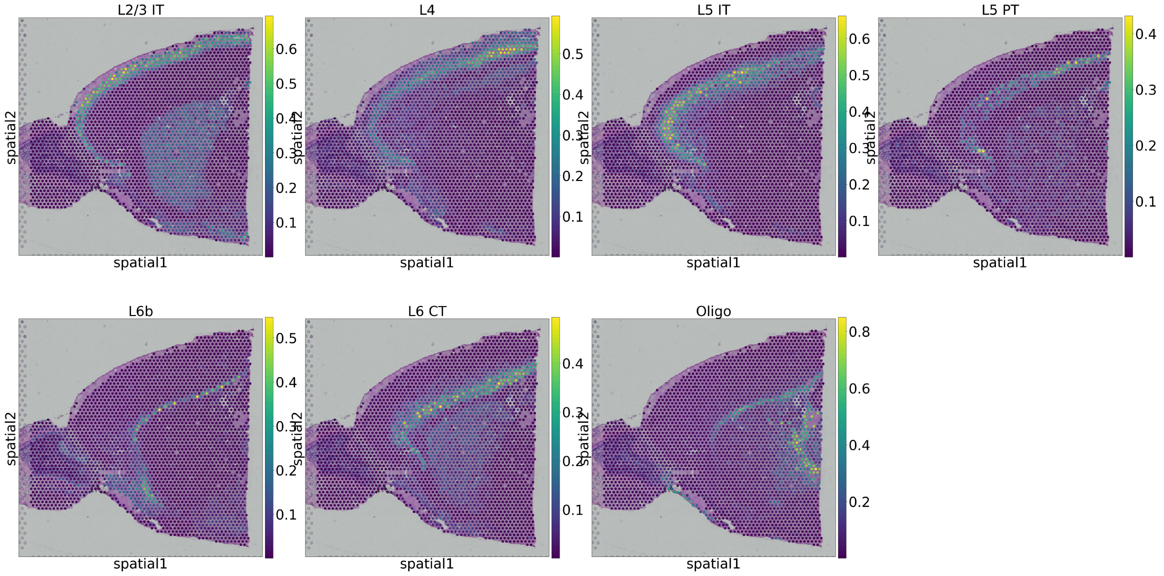

Supplementary Figure 8: Spatial mapping of major cell types in mouse cortex Visium dataset by GraphST tool.

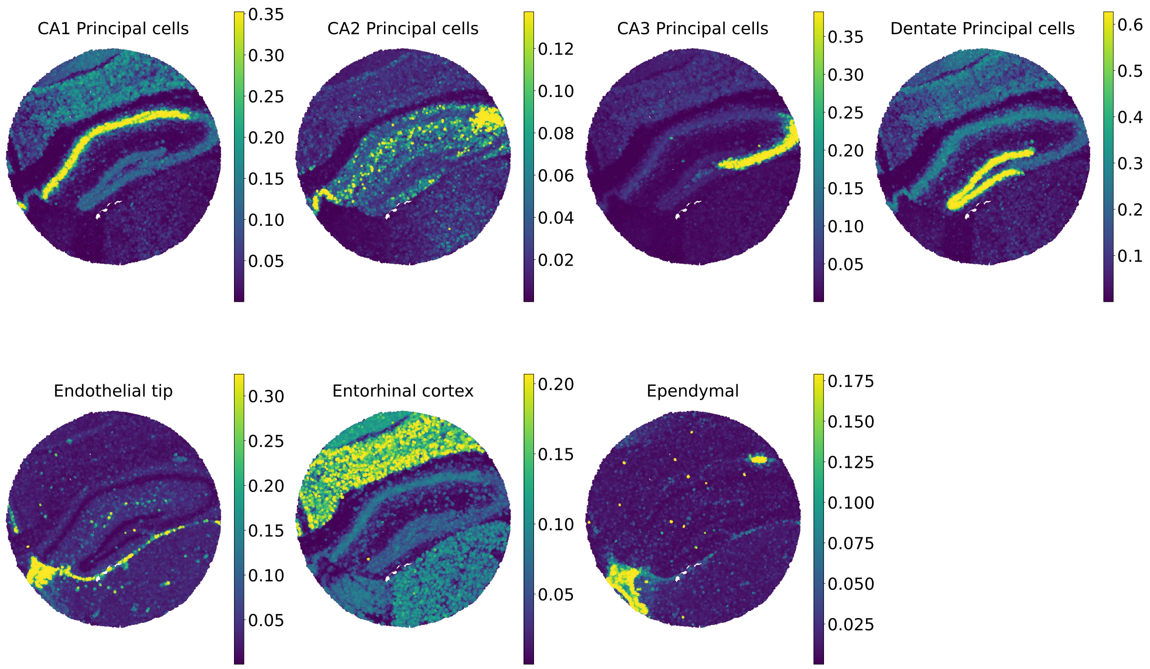

Supplementary Figure 9: Spatial mapping of major cell types in mouse Slide-seq hippocampus dataset by Cell2location tool.

Dataset type (Technology)	Tissue	Database	*Cells/Spots	Genes	#celltypes
Spatial (Visium)	Human cortex	LIBD	3,639	33,538	-
snRNA-seq	Human cortex	GEO	78,886	30,062	24
Spatial (Visium)	Mouse cortex	Visium	2,559	1,337	-
scRNA-seq	Mouse cortex	GEO	14,249	34,617	10
Spatial (slide-seq)	Mouse hippocampus	BISC-portal	53,173	23,264	-
scRNA-seq	Mouse hippocampus	BISC-portal	52,846	27,953	19
Spatial (Visium)	Mouse kidney	STOmicsDB	1,617	32,285	-
scRNA-seq	Mouse kidney	GEO	43,636	31,053	16
scRNA-seq	Mouse kidney	GEO	43,745	16,272	16
MERFISH spatial	Whole mouse	Allen Brain Atlas	1,19,927	500	-
STARmap spatial	Mouse cortex	STAGATE github	1207	1020	-

Supplementary Table 1: **Dataset type:** refers to either spatial or single-cell RNA-seq dataset; **Technology:** sequencing technology used to generate the dataset; **Tissue:** denotes the origin of tissue and species; cells/spots: indicate the number of cells for scRNA-seq dataset and number of spots for a spatial dataset; LIBD: Lieber Institute for Brain Development; BISC-portal: Broad Institute single cell portal; GEO: gene expression omnibus; **#celltype:** denotes the number of cell types present in the reference scRNA-seq datasets.
